# Associations between Quantitative Mobility Measures Derived from Components of Conventional Mobility Testing and Parkinsonian Gait in Older Adults

**DOI:** 10.1371/journal.pone.0086262

**Published:** 2014-01-22

**Authors:** Aron S. Buchman, Sue E. Leurgans, Aner Weiss, Veronique VanderHorst, Anat Mirelman, Robert Dawe, Lisa L. Barnes, Robert S. Wilson, Jeffrey M. Hausdorff, David A. Bennett

**Affiliations:** 1 Rush Alzheimer’s Disease Center, Rush University Medical Center, Chicago, Illinois, United States of America; 2 Department of Neurological Sciences, Rush University Medical Center, Chicago, Illinois, United States of America; 3 Laboratory for Gait and Neurodynamics, Movement Disorders Unit, Department of Neurology, Tel-Aviv Sourasky Medical Center, Tel-Aviv, Israel; 4 Department of Neurology, Beth Israel Deaconess Medical Center, Boston, Massachusetts, United States of America; 5 Harvard Medical School, Boston, Massachusetts, United States of America; 6 Department of Diagnostic Radiology and Nuclear Medicine, Rush University Medical Center, Chicago, Illinois, United States of America; 7 Behavioral Sciences, Rush University Medical Center, Chicago, Illinois, United States of America; 8 Department of Physical Therapy, Sackler Faculty of Medicine, and Sagol School of Neuroscience, Tel-Aviv University, Tel-Aviv, Israel; University of Glasgow, United Kingdom

## Abstract

**Objective:**

To provide objective measures which characterize mobility in older adults assessed in the community setting and to examine the extent to which these measures are associated with parkinsonian gait.

**Methods:**

During conventional mobility testing in the community-setting, 351 ambulatory non-demented Memory and Aging Project participants wore a belt with a whole body sensor that recorded both acceleration and angular velocity in 3 directions. We used measures derived from these recordings to quantify 5 subtasks including a) walking, b) transition from sit to stand, c) transition from stand to sit, d) turning and e) standing posture. Parkinsonian gait and other mild parkinsonian signs were assessed with a modified version of the original Unified Parkinson’s Disease Rating Scale (mUPDRS).

**Results:**

In a series of separate regression models which adjusted for age and sex, all 5 mobility subtask measures were associated with parkinsonian gait and accounted for 2% to 32% of its variance. When all 5 subtask measures were considered in a single model, backward elimination showed that measures of walking sit to stand and turning showed independent associations with parkinsonian gait and together accounted for more than 35% of its variance. Cross-validation using data from a 2^nd^ group of 258 older adults showed similar results. In similar analyses, only walking was associated with bradykinesia and sway with tremor.

**Interpretation:**

Quantitative mobility subtask measures vary in their associations with parkinsonian gait scores and other parkinsonian signs in older adults. Quantifying the different facets of mobility has the potential to facilitate the clinical characterization and understanding the biologic basis for impaired mobility in older adults.

## Introduction

Parkinsonian signs including unsteady and slow gait with balance and postural disturbances are common in older adults who do not have clinical Parkinson’s disease (PD) [1]. While these signs are robust clinical predictors of a wide range of adverse health outcomes, these signs lack specificity since many different disorders (i.e., neurologic, musculoskeletal, cardiopulmonary) can contribute to their development [1]–[3]. Furthermore the underlying CNS sites controlling these different signs and their underlying pathologic basis are unclear. Thus, while nigral pathology including Lewy bodies and neuronal loss is correlated with the severity of parkinsonism in older adults without clinical PD, recent work has shown that other common neuropathologies including Alzheimer’s disease and cerebrovascular disease are also associated with the severity of parkinsonism, especially parkinsonian gait[4]–[6]. This underscores the need for more specific mobility tests which have the potential to facilitate the clinical characterization and identification of the structural and pathologic basis underlying impaired mobility in older adults.

Mobility is not a unitary process but is derived from dissociable systems within the CNS which effect musculoskeletal structures to control its different features[7]. Laboratory investigations have quantified many subtasks of gait necessary for successful locomotion[8]–[10]. By contrast, a higher score on a modified version of the Unified Parkinson’s Disease Rating Scale (mUPDRS) indicating more severe impairment of parkinsonian gait does not specify which aspect of mobility is impaired. Similarly, prolonged time to complete different mobility performances does not specify which mobility subtask is impaired[11]. It is possible that different disease pathologies preferentially affect different subtasks leading to an overall higher mUPDRS score. Thus, quantification of the different components of mobility may inform, in future studies, on the pathologic basis of impaired mobility.

Rapid advances in technology have led to the availability of unobtrusive portable devices that which can record 3-dimensional movements and store large amounts of data for analyses at a later time. These devices offer the potential for obtaining a wide range of objective mobility measures in the community setting which, until recently, were only available in specialized laboratories. These measures can be obtained in the same amount of time as conventional testing and without additional burden to the individual being tested, while providing a more detailed characterization of mobility in older adults.

The overall goal of the current study was to quantify several mobility subtasks in community-dwelling older adults and examine which aspects of mobility are associated with the severity of parkinsonian gait. We used clinical data collected from 351 non-demented older adults participating in the Rush Memory and Aging Project to obtain more precise mobility measures in older adults tested in the community setting[12]. Prior studies have suggested that timed mobility performances are associated with parkinsonian gait[13]. Subjects underwent conventional gait testing in the community setting while wearing a belt with a small, light-weight whole body sensor which recorded both acceleration and angular velocity in 3 directions (DynaPortMiniMod Modules, McRoberts BV, the Netherlands). We derived gait measures from the sensor recordings that were used to quantify 5 mobility subtasks including: a) walking, b) transition from sit to stand, c) transition from stand to sit, d) turning and e) standing posture. The mUPDRS was used to assess the severity of parkinsonian gait and other signs. In a series of regression analyses, we examined the associations of the 5 quantitative mobility subtask measures which we derived alone and together with the severity of parkinsonian gait and other signs of parkinsonism.

## Methods

### Participants

All participants were from the Rush Memory and Aging Project, a longitudinal clinical-pathologic investigation of chronic conditions of old age that began in 1997[12]. Participants were recruited from retirement facilities and subsidized housing facilities from around the Chicago metropolitan area. All participants signed an informed consent agreeing to annual clinical evaluation and the study was in accordance with the latest version of the Declaration of Helsinki and was approved by the Institutional Review Board of Rush University Medical Center. Participants wore whole-body sensors (See below) starting in 2011. Persons were eligible for these analyses if they were ambulatory and did not have clinical dementia or PD when tested with these sensors.

### Clinical Assessment and Clinical Diagnoses

Participants underwent a uniform structured clinical evaluation each year that included a medical history, neurologic examination, and neuropsychological performance tests [14], [15]. Participants were evaluated by a physician who used all cognitive and clinical data to diagnose dementia and other common neurologic conditions as previously described [14], [15].

### Assessment of Parkinsonian Gait and Other Covariates

Trained nurses assessed 26-items from the motor section of the UPDRS. Four previously established scores for parkinsonism, including gait disturbance, bradykinesia, rigidity and tremor, were derived from these 26 items and a global parkinsonian score was based on their average[5]. A list of the items assessed, the approach used to summarize these data into individual parkinsonian signs and studies done to validate this instrument are provided as supplementary information (Appendix S1 and Table S1). Our primary outcome was parkinsonian gait. The parkinsonian sign scores had positively skewed distributions. The global parkinsonism and gait scores were subjected to a square root transformation, and the transformed scores were used as outcome variables in all analyses. Rigidity and tremor were relatively infrequent and so were treated as present or absent in analyses. Date of birth and sex were collected via participant interview.

### Assessment of Mobility

**Figure 1:**
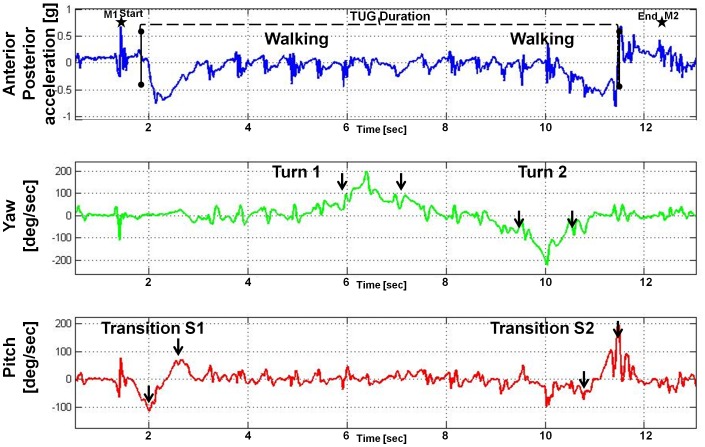
Timed Get Up and Go (TUG) Subtasks are Best Identified from Different Channels. This figure shows the acceleration and rotation signals recorded with a whole body sensor during conventional mobility testing of TUG in the community setting. The top channel shows the acceleration signal from the Anterior-Posterior (Blue) axis. The second channel shows the rotation signal of Yaw (Green, rotation around the vertical axis). Third, is the Pitch signal (Red, rotation around the mediolateral axis). The current study focused on several TUG subtasks including transition from sit to stand (S1), transition from stand to sit (S2), Turn 1 during the middle of the TUG and a second, Turn 2 which occurs immediately prior to sitting back down (S2). Walking measures can be extracted but in this study were derived from a 32 ft walk. To facilitate subsequent analyses, marks were inserted in the recorded data by the research assistant to identify the beginning and the end of each of the 3 performances analyzed in this study. The black star (M1) shows the first mark inserted when the research assistant pressed a button on the device immediately prior to instructing the participant to begin moving for the TUG. A second mark (M2) was inserted at the end when the task was completed. The M1 and M2 marks were used to extract the entire TUG trial from the continuous recording of the entire mobility testing session. After extraction of the entire TUG trial, an automatic algorithm was then applied for detecting the exact start and end times of the TUG based on the start time of the sit-to-stand (S1) and end time of the stand-to-sit (S2) AP signal (solid black line on AP channel). The Turn subtasks are visualized best from the Yaw (green) channel [black solid arrows Similarly, the Transition measures (S1 & S2) are best visualized on the AP (blue) and Pitch (red) channels [solid black arrows on the pitch]. Gait measures were derived from the onset and offset of the turns and transitions which are illustrated as described in the text (**Appendix S1**).

In an effort to minimize participant burden, we added whole body sensor recordings to the existing mobility testing protocol that has been used in the Memory and Aging Project since its inception. The 3 performances examined in this study comprise movements that are integral to mobility in older adults i.e. walking, standing posture, turning and transition from sit to stand and stand to sit. Participants were asked to walk an eight foot path back and forth twice without stopping for a total of 32 ft. Next, participants performed the Timed Up and Go (TUG) test twice (**Figure 1**). Participants were instructed to stand up from a chair, walk 8ft at a comfortable speed, turn and walk back to the chair and sit down again. Finally, the participants were asked to stand for 20 seconds with their eyes closed.

### Quantifying Mobility

#### Whole body sensor

During the conventional mobility testing described above, participants wore a portable small, light-weight whole-body sensor (Dynaport Hybrid, McRoberts, The Netherlands) on a neoprene belt placed on their lower back above the sacrum in the midline at the level of anterior iliac crest. The sensor weighs 74 grams and its dimensions are 87 × 45 × 14 mm. It includes a triaxial accelerometer (sensor range and resolution: ±2 g and 0.001 g, respectively) and a triaxial gyroscope (sensor range and resolution: ±100 deg/sec and 0.0069 deg/sec, respectively). The device recorded activity in three acceleration axes [vertical (V), mediolateral (ML), anterioposterior (AP)] and three angular velocity axes [yaw (rotation around the vertical axis); pitch (rotation around the mediolateral axis) and roll (rotation around the anterioposterior axis)].

#### Data collection

The device was set to record continuously during the entire conventional testing of mobility. The sequence of the tasks tested was the same for all participants. The data were saved on a secure digital card at a sampling frequency of 100 Hz. After testing, the data were reviewed by a research coordinator before being uploaded to a secure server for storage. The data were transferred to a personal computer for analyses at a later time (Matlab, version R2012b the MathWorks Inc, US).

#### Developing gait scores for analyses

We derived measures for a walking subtask from the 32ft walk since the longer distance allowed us to derive more robust walking measures. In prior work, we showed that the TUG has subtasks; for these analyses, we focus on: 1) transition from sit to stand, 2) transition from stand to sit, and 3) turning which are illustrated in **Figure 1** [16], [17].From the 20 second stand with eyes closed, we derived measures for a standing posture subtask. The research assistant pressed a button on the device to identify the beginning and end of each performance (**Figure 1**). The marks embedded in the recordings were used to segment the recordings and extract each performance alone. Automated algorithms were developed to derive a wide range of measures from each of the 3 performances recorded by the whole body sensor. Then we employed graphical and analytical techniques to analyze each of these performance and derived 31 gait measures from the 5 mobility subtasks as detailed in **Appendix S1** [16], [18]. Next, using principal component analyses and prior literature, we summarized these 31 measures into 13 gait summary scores. **Table 1** outlines the mobility performance decomposition and subsequent date reduction effort. The metric properties of these gait scores are described in **Appendix S1** and **Tables S2–S5**.

**Table 1:** Gait Measures and Gait Scores Derived from Whole Body Sensor Recordings Obtained during Conventional Mobility Performance Tests in the Community-Setting.

PERFORMANCE TESTS	MOBILITY SUBTASKS	GAIT MEASURES	GAIT SCORES
**32 ft Walk**	**Walk**	Speed (m/s)	**Speed**
		Stride length (m)	
		Cadence (steps/min)	**Cadence**
		Stride time CV (%)	**Variability**
		Stride regularity [g^2^]	**Regularity**
		Step symmetry	
**Timed Up & Go (TUG)**	**Sit to Stand (S1)**	AP Duration (s)	**Anterior-Posterior**
		AP Jerk (g/s)	
		AP range(g)	**Range**
		AP Acc SD (g)	
		Pitch range (deg/s)	
		Pitch jerk (deg/s^2^)	**Posterior**
		Median (deg/s)	
		Pitch Duration (g/s)	
	**Stand to Sit (S2)**	Pitch jerk (deg/s^2^)	**Jerk**
		AP duration (s)	
		Pitch duration (s)	
		AP Jerk (g/s)	
		AP range (g)	**Range**
		Pitch range (deg/s)	
		AP Acc SD (g)	
		Median (g)	**Median**
	**Turning**	Yaw, turn 1 (deg/s)	**Yaw**
		Yaw, turn 2 (deg/s)	
		Duration, turn 1 (s)	
		Duration, turn 2 (s)	
		Frequency, turn 1 (Hz)	**Frequency**
		Frequency, turn 2 (Hz)	
**Standing With Eyes Closed**	**Standing Posture**	Jerk [g/s]^2^	**Sway**
		RMS distance [g]	
		Total power [psd]	

### Statistical Analysis

The goal of these analyses was to examine the contributions of different mobility subtasks to parkinsonian gait. Our approach consisted of 2 stages which employed a series of multiple regression models. In the first stage, we used gait scores to derive an outcome specific score for each of the 5 mobility subtasks. In the 2nd stage, we used the 5 individual mobility subtask scores which we derived to determine which subtasks showed independent associations with parkinsonian gait and other parkinsonian signs. For outcomes that had enough variation to be analyzed as continuous variables (parkinsonian gait, global parkinsonian score), we used linear regression analyses. For measures that were less common and not observed in many participants (bradykinesia and rigidity), we used logistic regression models of binary outcomes i.e., the presence or absence of these signs. We describe our methods for the analysis of our primary outcome measure parkinsonian gait in detail. Similar analyses were conducted for the other signs of parkinsonism and for the global parkinsonian score. A more detailed description of these analyses is included in **Appendix S1**.

First, we examined a series of regression models and discarded scores not associated with parkinsonian gait. In the second step, we included all the gait scores which were associated with parkinsonian gait in a single model and employed backwards elimination. We used standard selection criteria for the backwards elimination steps: p-to- remove was set at the default alpha = 0.10.This identified the best model for which each of the subtasks constituent scores showed separate effects with parkinsonian gait. For example, 3 of 4 individual walking scores (cadence, speed and regularity) were associated with parkinsonian gait (results not shown). After backward elimination, speed and regularity remained associated with parkinsonian gait (**Table 2**). We used the regression coefficients for all significant terms in the final model to compute a fitted subtask measure for parkinsonian gait. Since backwards elimination can eliminate important variables early in the process in the presence of correlated predictors, we also reviewed all subsets of the predictors to avoid anomalies and ensure that the model we selected was appropriate.

**Table 2:** Regression Coefficients for Gait Scores Used to Compute Fitted Mobility Subtask Scores for Parkinsonian Signs (Stage 1).

Mobility Subtasks	Gait Scores	Parkinsonian Gait	Bradykinesia	Tremor	Global Park
**Walk**	**Speed**	−0.466	2.718	–	−0.470
	**Cadence**	–	–	–	–
	**Variability**	–	–	–	–
	**Regularity**	−0.152	–	–	−0.118
**Sit to Stand (S1)**	**Anterior-Posterior (S1)**	–	–	–	–
	**Range (S1)**	–	–	–	–
	**Posterior (S1)**	−0.427	–		–0.376
**Stand to Sit (S2)**	**Jerk (S2)**	−0.208	–	–	−0.236
	**Range (S2)**	–	–	–	–
	**Median (S2)**	–	–	–	–
**Turning**	**Yaw**	−0.615	–	–	−0.586
	**Frequency**	–	–	–	–
**Standing Posture**	**Sway**	−0.146	–	2.718	−0.204

In the second stage of these analyses, we examined the contributions of the 5 fitted subtask scores to parkinsonian gait. The approach employed in this stage was similar to the approach described above. In a first step, we examined the contributions of each of the 5 adjusted subtask measures alone with parkinsonian gait as the outcome. We eliminated any subtask that was not associated with the parkinisonian gait outcome. Then we employed backward elimination to determine which subtasks measures showed separate effects with parkinsonian gait when they were considered together. Overfitting can occur when automatic methods are used and may exaggerate measures of agreement. However the process will not be biased to favor certain predictors over others. To validate this analytic approach, a cross-validation study was performed in a second group of participants who had undergone similar mobility testing.

We used a similar approach to examine the contributions of the 5 subtask measures with bradykinesia and tremor, as well as a global summary of parkinsonian signs. In the analyses of bradykinesia and tremor, we used logistic regression for presence versus absence of the parkinsonian sign when the parkinsonian sign was seen in less than 60% of this group of participants. Since it is not appropriate to adjust the binary outcome directly, we used the estimated person-specific logit from the model with age and sex only as an offset term (entered with coefficient forced to be 1 in all the other models). Rigidity was too infrequent (N = 16, 4.6%) to allow for meaningful analyses at the present time.

For all steps described above, models were examined graphically and analytically and assumptions were judged to be adequately met. Graphical and analytic review included checks of standard statistical diagnostics (Cook’s D, influential points, residuals, residual plots, and checking correlations among predictors). *A priori* level of statistical significance was 0.05. Programming was done in SAS version 9.3 (SAS Institute Inc, Cary, NC)[19].

## Results

These analyses were based on 351 participants whose clinical characteristics are summarized in **Table 3**.

**Table 3:** Characteristics of Participants (N = 351).

Variable	Mean (SD) or N %
Age (yrs)	78.8 (6.74)
Sex (women)	275 (78.4%)
Education (yrs)	15.0 (2.80)
BMI (kg/m2)	27.1 (5.21)
Mini Mental Status Examination	27.6 (3.12)
**Parkinsonian Scores (0–100)**	
Global Parkinsonism	5.6 (5.38)
Parkinsonian gait	12.9 (13.16)
Rigidity score	0.38 (1.94)
Tremor score	1.8 (4.72)
Bradykinesia score	7.3 (9.74)
**Parkinsonian trait Present (N, %)**	
Any	299 (85.2%)
Parkinsonian gait	257 (73.2%)
Rigidity	16 (4.6%)
Tremor	77 (21.9%)
Bradykinesia	196 (55.8%)

### Quantitative Mobility Subtask Measures and Parkinsonian Gait

Our primary analysis examined the contributions alone and together of 5 mobility subtasks with parkinsonian gait. In the first step, we use the fitted values for each of the 5 subtasks as predictors in multiple regression models (**Table 4** **, Step 1, Models A–E**) to examine the contributions of the 5 subtasks alone with parkinsonian gait. For this reason, the single regression coefficients for models A-E are all equal to 1. Each column corresponds to a regression of adjusted parkinsonian gait score for a different subtask measure. The Adj-R-sq is the fraction of variation of parkinsonian gait explained by the subtask score relative to the variation not explained by demographic terms. All 5 subtasks were associated with parkinsonian gait. Walking, turning or transition from sit to stand each accounted for 20% or more of the variance of parkinsonian gait. By contrast, the sway and transition from stand to sit measures accounted for 2% and 5% of the variance.

**Table 4:** Quantitative Mobility Subtask Measures and Parkinsonian Gait (Stage 2).

	STEP 1 Linear regression models					STEP 2 Backward elimination	
Mobility Subtasks	Model A β(SE, p-Value)	Model B β(SE, p-Value)	Model C β(SE, p-Value)	Model D β(SE, p-Value)	Model E β(SE, p-Value)	Model 1 β(SE, p-Value)	Model 2 β(SE, p-Value)
**Adj R-Sq**	0.319	0.197	0.047	0.327	0.022	0.352	0.353
**Walk**	1.000 (0.109,<0.001)					0.418 (0.167,0.013)	0.418 (0.164, 0.012)
**Sit-Stand (S1)**		1.000 (0.163,<0.001)				0.455 (0.178,0.012)	0.399 (0.172, 0.022)
**Stand-Sit (S2)**			1.000 (0.341,0.004)			−0.414 (0.339,0.225)	
**Turning**				1.000 (0.117,<0.001)		0.540 (0.173,0.002)	0.510 (0.172, 0.004)
**Standing** **Posture**					1.000 (0.441,0.025)	0.334 (0.452,0.461)	

This table shows the final step of a multistage process which was used to develop 5 quantitative mobility subtask measures from whole body sensor recordings and to examine their associations with parkinsonian gait score. On the left is a series of linear regressions to determine which of the 5 quantitative mobility subtask scores were associated with parkinsonian gait score. Each cell shows the **β** coefficients from the regression for the terms included, with (Standard Error, p-value) below. The Adj-R-sq is the adjusted R^2^ with the adjusted parkinsonian score, that is the fraction of variation explained relative to the variation of parkinsonian score not explained by demographic terms. By construction of the subtask scores, the single regression coefficients for models 1–5 are all equal to 1. In a second stage (right 2 columns), we employed backward elimination and started with a backward elimination regression model (Model 1) that included all 5 subtask scores which were all associated with parkinsonian gait score when considered alone in models A–E. Two subtasks did not show significant independent associations with parkinsonian gait; neither of these two subtasks were retained in the final model which showed that walking, turning and sit to stand accounted for 35% of the variance of adjusted parkinsonian gait.

In the next step, we used a backwards elimination regression algorithm (**Table 4** **, Step 2, Model 1**) which included all 5 subtask measures which had been associated with parkinsonian gait in step 1 (**Table 4** **, A–E**). Model 2 (**Table 4**) was selected by backwards elimination since all the gait subtask measures included show separate effects with adjusted parkinsonian gait score. When considered together, the subtask measures for walking, sit to stand and turning showed independent associations with adjusted parkinsonian gait and accounted for more than 35% of the residual variance of adjusted parkinsonian gait (Model 2). The most robust predictor was turning.

To cross-validate our results and address concerns about overfitting, we repeated these same analyses in a 2^nd^ group of 258 additional MAP participants who had undergone the same mobility testing. The same 3 subtasks (walk, sit to stand and turns) showed independent associations with parkinsonian gait and accounted for 32% of the residual variance of adjusted parkinsonian gait. These data are included in **Table S6** and discussed in **Appendix S1**.

### Quantitative Mobility Subtask Measures and Other Parkinsonian Signs

Parkinsonian gait is one of four cardinal motoric signs of parkinsonism. We next examined the extent to which the 5 subtasks were associated with other parkinsonian signs. The multistage process described above was not necessary since only the walking subtask was associated with bradykinesia and the sway subtask measure was associated with tremor (**Table 2**). In a final set of analyses, we examined the association of the 5 subtask measures, with global parkinsonism, a summary measure based on all 4 parkinsonian signs. When considered individually, all 5 subtask measures were associated with global parkinsonism and accounted for between 4% to 30% of the variance. In a model selected by backwards elimination the quantitative subtask measures for walking and turning showed independent associations with adjusted global parkinsonism accounting for almost 30% of the residual adjusted variance of global parkinsonism.

## Discussion

This study obtained a more detailed clinical characterization of mobility in 350 ambulatory undemented older adults who wore a whole body sensor while undergoing conventional mobility testing in the community-setting. Measures derived from these recordings were used to quantify 5 previously identified mobility subtasks. These subtasks included walking, transition from sit to stand, transition from stand to sit, turning, and standing posture. Results showed that while all 5 subtasks were associated with parkinsonian gait, there was a wide disparity in the variance of parkinsonian gait accounted for by each of the individual subtasks (2%–32%). Further, only walking, transition from sit to stand and turning showed independent associations when the 5 subtasks were considered together. Using this analytic approach, we obtained similar results in a second group of 258 older adults who underwent identical mobility testing. Of the 5 subtasks, only walking was associated with bradykinesia and only sway was associated with tremor[20]. Quantifying the different facets of mobility with a whole body sensor during conventional mobility testing has the potential to provide more detailed characterization of impaired mobility in older adults without additional testing burden.

Prior laboratory studies suggest that quantitative measures of mobility obtained during traditional gait testing can increase the detection of gait impairments and the prediction of adverse health outcomes such as dementia and falls[16], [17], [21]–[27]. The current study extends these laboratory-based studies in several important ways. First, the device employed in the current study measured both acceleration and angular velocity signals for three orthogonal axes. Measuring both simultaneously allowed us to derive both quantitative spatiotemporal measures of gait, as well as rotation and tilt which occur during the testing of mobility. Second, the current study shows that it is now feasible to incorporate an unobtrusive whole-body sensor during conventional mobility testing. This approach allows investigators to continue longitudinal data collection of traditional gait metrics, as well as providing novel quantitative measures on the full spectrum of mobility among older adults living in the community setting. Third, in contrast to prior studies, this study collected quantitative measures for several mobility subtasks from older adults without overt neurologic diseases and developed an analytic approach which was used to examine their contributions to parkinsonian gait and other parkinsonian signs.

Emerging technologies that are used to study mobility can be used to derive numerous objective measures from a single performance and methods are needed to reduce and summarize these data. Several recent studies have described approaches for reducing multiple measures obtained from recordings of a single walking task into 2 or more factors[21], [22]. The current study employed a similar approach for reducing multiple measures to 1 or more scores for each of the 5 subtasks. Since we hypothesized that the contributions of these different subtasks would vary with different outcomes and since the subtasks are not themselves highly correlated, we did not create a single summary measure for all the subtasks together, nor did we employ a fixed summary measure for each individual subtask. Rather, we chose to compute fitted values for each subtask for different outcomes which we examined. Consistent with this idea, our analyses showed that the associations of the 5 subtasks varied substantially across the parkinsonian signs that we examined (i.e. parkinsonian gait, bradykinesia and tremor). Furthermore, the additive associations of different subtasks with parkinsonian gait underscores the importance of testing several subtasks to obtain a more comprehensive description of the facets of mobility associated with diverse outcomes. Similarly, the lack of association of transition from standing to sit and sway with parkinsonian gait suggests that these subtask measures may capture additional features of mobility or other motor behaviors (i.e. strength, balance) not assessed by the UPDRS[18], [28]. Finally, the approach employed in the current study could be expanded to a wider range of mobility performances in order to develop a more complete inventory of mobility subtasks for explicating the pathologic basis for impaired mobility in older adults.

The notion that the control of varied clinical mobility subtasks derives from distinct CNS neural networks has the potential to transform the clinical categorization of impaired mobility in older adults. Rapid advances in imaging and neurophysiologic methods have led to increasing evidence about the complexity of neural mechanisms which underlie mobility, but these advances have not been fully assimilated into the clinical domain. Mobility occurs in three-dimensional space and requires the production of coordinated rhythmic activations of both legs and the trunk as well as the postural control of the moving body which are adapted to self-motivations and environmental demands. Localized brain lesions (e.g., stroke), specific diseases (e.g., Parkinson’s disease, radiculopathy, or myositis), and localized musculoskeletal disease (e.g., osteoarthritis) may selectively impair distinct aspects of mobility while sparing others[29]–[31]. These dissociations suggest that mobility is not a unitary process and that the clinical manifestations of impaired mobility vary with the localization of CNS and musculoskeletal systems dysfunction[32]–[35].

These clinical observations are supported by recent advances which have begun to characterize the neural processes that underlie mobility. Integration of a wide range of sensory and visuospatial information is essential for intact gait [i.e. postural control, spatial navigation, and joint position][36] and different regions within mobility related brain regions may control distinct aspects of gait [i.e., speed versus balance][37]. Finally, brain structures outside traditional motor regions are also crucial for gait with increasing evidence from animal studies for the importance of brainstem and spinal cord locomotor circuits[38]–[42]. Translating these basic science observations into the clinical domain is essential but requires an expansion of the range of clinical measures which are obtained routinely during conventional mobility testing. While clinical instruments like the mUPDRS are robust clinical predictors of adverse health outcomes, CNS control of the individual items which are assessed is unclear. Even for subspecialties which focus on localizing specific CNS lesions with varied locomotor abnormalities, objective assessment of mobility subtasks remains a research tool. The current study suggests that it is now feasible to incorporate devices which can provide a wide range of objective mobility measures. Further work will be necessary to delineate the specificity and sensitivity of these mobility subtask measures, compare their concurrent validity with other devices and delineate their underlying structural basis. This approach offers the potential for more accurate clinical characterization of mobility and identification of the localization and pathologic basis underlying impaired mobility in older adults. This in turn would provide a host of new targets for interventions to meet this growing public health challenge.

The current study has several limitations. We used a volunteer cohort of community-dwelling adults who may not be representative of the general population, so the results need to be confirmed in other cohorts. These analyses were cross-sectional and longitudinal studies are needed to separate change from cohort effect. The current study examined only 3 mobility tasks that are not likely to exhaust all the subtasks which may contribute to impaired mobility in older adults. As our understanding of mobility increases, additional subtasks can be added to provide investigators and clinicians the means to more fully characterize impaired mobility in older adults. The strengths of the present study include the use of a device that measured both acceleration and angular velocity in 3 planes to quantify several subtasks commonly used to evaluate mobility and examined a large number of men and women without clinical dementia in the community-setting.

## Supporting Information

Appendix S1 **Supplemental Methods.**
(DOCX)

Table S1 **Modified UPDRS Assessment.**
(DOCX)

Table S2 **Gait Measures and Gait Scores Derived from Whole Body Sensor Recordings.**
(DOCX)

Table S3 **Association of Gait Scores with Age and Sex.**
(DOCX)

Table S4 **Correlations of Walking, Turning and Sway Gait Scores.**
(DOCX)

Table S5 **Correlations of Transition Scores, Turning and Sway Gait Scores.**
(DOCX)

Table S6 **Cross Validation of the Original Results in a 2nd Group of Older Adults (N = 258).**
(DOCX)
