# Supplementary material for: Associations between Quantitative Mobility Measures Derived from Components of Conventional Mobility Testing and Parkinsonian Gait in Older Adults
Source: PLoS One. 2014 Jan 22;9(1):e86262. doi: 10.1371/journal.pone.0086262 (PMC3899223; doi:10.1371/journal.pone.0086262)
Supplement: Appendix S1 — Supplemental Methods. (DOCX) [file pone.0086262.s001.docx]

**APPENDIX S1. SUPPLEMENTAL METHODS**

**1. ASSESSMENT OF PARKINSONIAN SIGNS**

Trained nurse clinicians administered a modified version of the motor portion of the United Parkinson’s Disease Rating Scale (mUPDRS). The modifications were minor and were intended to make the scale more applicable to persons without PD and easier for non-physicians to administer and score.[[1](#_ENREF_1),[2](#_ENREF_2)] There were 26 items which were examined to assess 4 parkinsonian signs (Table 1). *Parkinsonian gait* was based on 6 items: arising from a chair, shuffling gait, body bradykinesia, turning, posture, and postural stability. *Rigidity* was based on 5 items, one each for neck and all four extremities. *Bradykinesia* was based on 8 items: right and left finger taps, fist clench, pronation-supination, and leg agility. *Tremor* was based on 7 items: resting tremor of chin-jaw and all four extremities, and action-postural tremor of both hands. Each of the 26 items was rated on a similar 0-5 scale: 0=Normal; 1=Slowing or reduction in amplitude which could be normal; 2= Mild slowing and reduction in amplitude; 3=Moderately impaired, definite early fatiguing or may have occasional arrests in movement; 4=Severely impaired. Frequent hesitation in initiating movements; 5=Can barely perform the task.

Each of the 4 parkinsonian sign scores was calculated by adding the number of points (0-5) assigned to each of the individual items which were rated e.g., 8 items for bradykinesia. The total number of points for these 8 items is the raw bradykinesia score. This score was then divided by the maximal possible score which could be given for the domain which in the case of bradykinesia is 40 (8x5=40). This ratio (actual points/maximal points) was then multiplied by 100. Thus, while the number of items varied for each of the 4 parkinsonian signs, each the four signs were scaled from 0 to 100. A summary global parkinsonian sign score was constructed by averaging the four individual parkinsonian sign scores. These 5 measures of parkinsonism which we constructed have high inter-rater reliability and short-term temporal stability and are reproducible in men and women in aging and dementia from a variety of cohorts[[1](#_ENREF_1),[2](#_ENREF_2)].

A series of studies in this and other cohorts have shown that the severity of parkinsonism in older adults is related to incident disability, falls, AD and survival as well as the rate of cognitive decline.[[3-8](#_ENREF_3)] While these signs are robust clinical predictors of a wide range of adverse health outcomes, these signs lack specificity since many neurologic as well as non-neurologic disorders (i.e., musculoskeletal, cardiopulmonary) can contribute to their development. Furthermore, the underlying CNS sites controlling these different signs are unclear and these signs lack specificity with respect to their underlying pathologic basis[[3](#_ENREF_3),[8](#_ENREF_8),[9](#_ENREF_9)]. Thus, while nigral pathology including Lewy bodies and neuronal loss is correlated with the severity of parkinsonism proximate to death in older adults without clinical PD, recent work has shown that other common neuropathologies including Alzheimer’s disease and cerebrovascular disease as well as neuronal loss are also associated with the severity of parkinsonism, especially parkinsonian gait[[10-12](#_ENREF_10)]. This underscores the need for more specific mobility tests which will facilitate efforts to identify the location and pathologic basis underlying late-life gait impairment.

**2. QUANTIFYING MOBILITY SUBTASKS USING WHOLE BODY SENSOR RECORDINGS**

1. **Processing Whole Body Sensor Recordings**

During testing in the community, the research assistant doing the testing, pressed a button on the Dynaport device which inserted a mark in the whole body sensor recording a the beginning and end of each individual performance. This is illustrated in **Figure 1** for the TUG. Each task was then processed to extract various gait measures as described below.

***Performance 1. Walking Performance***

*Processing for Walking Measures:* All acceleration measures were calculated based on the vertical acceleration axis. Evans et al showed that the vertical axis acceleration provides important information about the gait cycle and is the most sensitive for gait disorders in the assessment of gait[[13](#_ENREF_13)]. The acceleration signal was calibrated using Moe Nilssen’s calibration algorithm, which transforms the data to the horizontal-vertical coordinate system[[14](#_ENREF_14)]. The signal was then normalized by subtracting the mean and dividing by the standard-deviation. The temporal acceleration measures were calculated by high pass filtering the acceleration signal at 0.2 Hz, integrating, and detecting the peaks (local minima and maxima). The inter-peak intervals gave an estimate of the step time. The intervals between every second peak determined the stride times. An automated filtering procedure was applied to eliminate outliers with respect to the mean of the stride time series[[15-18](#_ENREF_15)].

**Walking Measures**

| Walking speed (m/s) | Distance =32ft/time to complete task (converted to m/s) |
| --- | --- |
| Stride length (m) | 32ft/number of strides (converted to meters) |
| Cadence | Number of steps/min |
| Stride time CV (%) | This is a measure of gait variability. The coefficient of variation of stride time series [%] (=100* stride time standard deviation/average stride time). |
| Stride regularity (g^2^) | Extracted from the time domain; autocorrelation signal.[[14](#_ENREF_14)] |
| Step symmetry | Step regularity/stride regularity |

***Performance 2. Timed Up and Go Task***

An automated algorithm for detecting the start and end times of the TUG based on the anterior-posterior axis was used to derive the overall time that it took for the participant to complete the TUG, as previously described[[19](#_ENREF_19),[20](#_ENREF_20)]. In addition, quantitative measures for 4 subtasks (transition from sit to stand, transition from stand to sit, walking and turning) were derived[[19](#_ENREF_19),[20](#_ENREF_20)].

*Processing for Transition Measures:* The same 8 measures were extracted for transition from sit-to-stand (S1) and stand-to-sit (S2) from the anterior-posterior (AP), vertical (V) and pitch (P) axes (see **Figure 1**). These measures were developed in prior publications and included: the duration of the task, the range of motion, jerk measures, median and standard deviation values (see Figure 1).[[19-21](#_ENREF_19)] The duration of the transition was based on AP axis. Jerk was determined as the estimated slope of the AP or pitch signals between the maximum and minimum points[[19-21](#_ENREF_19)].

**Transition Measures (Sit to Stand and Stand to Sit)**

| AP Duration (s) | Duration of transition interval based on anterior-posterior (AP) axis |
| --- | --- |
| AP range(g) | Acceleration range during the transition interval (AP axis) |
| AP Jerk (g/s) | Slope during the transition interval (AP axis) derivative, |
| AP Acceleration SD (g) | Standard deviation of AP axis acceleration (g) |
| Pitch Duration (s) | Duration of transition interval – pitch axis (mediolateral rotation [ML]) |
| Pitch range (deg/s) | Angular velocity range during the transition interval based on pitch axis |
| Pitch jerk (deg/s^2^) | Slope during the transition interval (pitch axis) |
| Median (deg/s) | Median of the angular velocity amplitude (pitch axis) |

*Processing for Turning Measures:* Turning measures were derived from the yaw axis. These subtasks appear as two large amplitude peaks in the signal (**Figure 1**). The 1^st^ peak represents the turn performed in the middle of the TUG, i.e., turn-to-walk, and the 2^nd^ peak represents the turn performed at the end of the trial, before the second transition from standing to sitting. The start and end points of each separate turn were determined as the points in the yaw signal in which it crossed 0.1 of the maximum yaw peak amplitude of the turn. Turn duration was determined as the duration from the beginning until the end of the turn[[19-21](#_ENREF_19)].

**Turning Measures**

| Yaw amplitude, trial 1 (deg/s) | Amplitude of angular velocity (rotation) around the vertical axis (V) |
| --- | --- |
| Yaw amplitude, trial 2 (deg/s) | Amplitude of angular velocity (rotation) around the vertical axis (V) |
| Duration, trial 1 (s) | Duration of turn |
| Duration, trial 2 (s) | Duration of turn |
| Frequency, trial 1 (Hz) | Acceleration frequency during turn |
| Frequency, trial 2 (Hz) | Acceleration frequency during turn |

***Performance 3. Standing Posture***

*Processing for Standing Posture Measures:* All sway measures were derived from a minimal common duration of 17s trial length (to eliminate deviations caused by trial duration). Jerk is the time derivative of acceleration, and quantifies the amount of active postural corrections during quiet standing and is dependent on the signal size. In order to calculate the jerk, the raw acceleration signal was filtered to account for possible contributions of tremor at rest. We used a 3.5 Hz cutoff, zero-phase, low pass, Butterworth filter. The root mean square of acceleration (RMS), quantifies the magnitude of the AP, ML and RD. RD, the resultant distance, is calculated as the square root of the sum of the squares of the AP and ML[[22-24](#_ENREF_22)]. The total power is derived from the same filtered signal from which the Jerk is derived. Total power is the integrated area of the power spectrum for AP, ML and RD[[22](#_ENREF_22),[23](#_ENREF_23)].

**Standing Posture Measures**

| Jerk [g/s]^2^ | Derivative of acceleration, measure of smoothness of postural corrections |
| --- | --- |
| RMS distance [g] | Root mean square quantifies the magnitude of acceleration |
| Total power [psd] | Integrated power spectrum for all 3 axis |

1. **Deriving Gait Measures**

Descriptive statistics and plots were used to examine the distributions and heterogeneity of the measures derived from the whole body sensor data. We reduced the number of gait measures by using scatterplot matrices of individual measures to exclude those that are highly correlated with other measures selected and those measures demonstrating extreme outliers. Measures that were highly correlated with other selected measures were set aside at this stage. Some measures were skewed unless transformed: in these cases, logarithmic transformation decreased skewness substantially, and improved symmetry of distributions.

The focus of our studies is understanding variation between individuals, underscoring the importance of identifying quantitative measures that are stable within-person (trial to trial variation) while varying between individuals. The TUG was repeated so there were replications of each of the 2 transitions (S1 and S2) and 2 turns (Turn 1 and Turn 2) as illustrated in **Figure 1**. We examined components of variance due to trial and person for each of the 4 subtasks (S1, S2, Turn 1 and Turn2). For most measures we retained, the person-to-person variation is larger than trial to trial variation within each of the individual subtasks. Next, we examined whether it was justified to combine similar subtasks (S1 and S2; Turn 1 and Turn 2). We combined the similar subtasks and again examined the components of variance due to trial and person. For example, considering Yaw and Duration measures from the Turn subtasks, we found that the two turns were so similar within person (less than half of the trial-to-trial variation). In contrast, when we examined the transition subtasks (S1 and S2), 7 of the 8 measures showed more than half of the trial to trial variation. Moreover, principal components analyses captured higher within-transition subtask correlation than between transition correlation, whereas the principal components of the turn subtasks (Turn 1 and Turn 2) showed a single across-component subtask. The components of variance analyses and the principal components analyses led us to combine Turn 1 and Turn 2 into a single Turning subtask. In contrast, these analyses justified separate analyses for the S1 and S2 subtasks.

At the end of this first stage, there were 31 measures which we had derived from the five subtasks (**Table S2**). These included 6 measures to quantify walking. We derived 8 measures for each of the 2 transition subtasks and 6 measures to quantify the turning subtask from the TUG test (**Figure 1**). Finally, we derived 3 measures based on the standing posture subtask (Figure 1).[[21](#_ENREF_21),[25](#_ENREF_25)] Descriptive statistics showed heterogeneity of the gait measures (**Table S2**). We then used linear regression to examine the associations of the gait measures with age and sex.

1. **Deriving Gait Scores**

We used prior literature and principal component analyses to summarize the 31 measures into 13 scores. Each score had a standard deviation equal to 1, and higher values corresponded to more movement. The scores were grouped by the subtasks from which they were derived. Next we obtained age- and sex-adjusted values for each score. Separately for each of the gait scores, obtained residuals from regression on age and sex. Each score was scaled to have mean 0 and SD = 1.The partial correlations of the scores contributing to each of the 5 subtasks were reviewed. To facilitate comparisons, all 13 adjusted scores were centered at the means of the original variables and scaled by dividing by their standard deviations.

Descriptive statistics showed heterogeneity of the scores (**Table S2**). Most scores showed some association with age or sex (**Table S3**). The 4 scores which had adjusted R^2^ greater than 0.10 [speed, regularity, posterior (S1), yaw] showed strong associations with age. Speed and regularity were also associated with sex. An additional 4 scores showed modest association with age [variability, anterior-posterior (S1), range (S1), sway]. Sway and jerk (S2) were related to sex. The 4 remaining scores [cadence, range (S2), median (S2), and frequency] were not related to age or sex. Then we adjusted the scores for age and sex so we could directly compare the contributions of the gait scores to parkinsonian gait. The correlations between the scores and the partial correlations adjusted scores are shown in **Table S4** and **Table S5**. Partial correlations between scores within each of the 5 subtasks were always below 0.0.50 in absolute value, so that any score explains less than 25% of the variation of another score. Often these correlations were less than 0.1 in absolute value, indicating that the measures were not importantly correlated, suggesting that these variables capture different mobility constructs. Some scores from different subtasks showed substantial correlations. For example the partial correlation of the yaw measure exceeded 0.3 in absolute value with speed, regularity, anterior-posterior (S1), range (S1), posterior (S1), and jerk (S2). These gait scores were used to develop outcome specific scores for each of the 5 mobility subtasks in the manuscript as described more fully below.

1. **Deriving Subtask Scores and Examining their Contribution to Parkinsonism**

The primary outcome in this study was parkinsonian gait, but there were 3 other outcomes including: global parkinsonian score, bradykinesia and rigidity. Parkinsonian gait and global parkinsonism: We used the square-root transformation of the score . The overall goal of our analyses was to determine which of the 5 mobility subtasks showed independent associations with parkinsonian gait and other parkinsonian signs.To directly compare these 5 subtasks in joint models, we developed an approach through which we could derive a single score for each of the 5 mobility subtasks. Our approach consisted of 2 stages which employed series of multiple regression models. In the first stage, we used gait scores to derive an outcome specific score for each of the 5 mobility subtasks (**Table 4**). In the 2^nd^ stage, we used these 5 individual mobility subtask scores to determine which subtasks showed independent associations with parkinsonian gait and other parkinsonian signs. For outcomes that had enough variation to be analyzed as continuous variables (parkinsonian gait, global parkinsonian score), we used regular regression analyses. For measures of phenomena that were less common and not observed in many participants (bradykinesia and rigidity), we used logistic regression models of binary outcomes i.e., the presence or absence of these signs.

*Stage 1: Deriving Subtask Mobility Scores:* We sought to obtain a single outcome specific score for each of the 5 mobility subtasks. In the first step of this stage we used the gait scores described above for each of the 5 mobility subtasks. We analyzed each of the 5 subtasks separately using a systematic set of multiple regression models to determine which gait scores for each mobility subtask showed independent associations with each of the parkinsonian signs and global parkinsonian score .

For example, for parkinsonian gait, which could be analyzed as a continuous variable, we used linear regression models. We obtained residuals which were centered and scaled to mean 0 and SD=1 from regression of the square-root transformed parkinsonian gait score on age and sex. Then we regressed parkinsonian gait (adjusted for age and sex) on each of the adjusted gait scores alone for each of the 5 mobility subtasks. If p> 0.05, did not carry this gait score forward. If no gait score had p<0.05, then we did not retain any measures for this subtask. Otherwise we moved to the 2nd step which employed backward elimination, starting with those gait scores not eliminated in the first stage. We used standard backwards elimination (p-to-remove was 0.10). Using the regression model obtained when the backward elimination stopped, we calculated the fitted values for parkinsonian gait for each of the subtasks using the coefficients derived in Stage 1 and shown in **Table 2**.

*Stage 2: Mobility Subtask Scores & Parkinsonism:* In this stage we used the 5 fitted mobility subtask scores developed in Stage 1, in a 2^nd^ series of regression models, to examine which subtasks showed independent associations with parkinsonian gait and the other parkinsonian signs. For example, in the first step of Stage 2, we regressed parkinsonian gait on the fitted value for each of the 5 subtasks alone (**Table 4, Step 1, Models A-E**). When a single constructed variable is considered for the linear regression models, the estimated coefficient in the linear model fit is 1. This step provides a check of our work, in that the regression coefficient must be 1 if our prior calculations are correct (**Table 2)**. In the first step of Stage 2, if we found a subtask with p>0.05, that subtask was dropped. In step 2 of Stage 2, backwards elimination was again employed starting with those subtasks with p<0.05 (**Table 4, Step 2, Model 1**). Terms for all such subtasks are considered simultaneously in Model 1, with the model selected by backwards elimination (again, standard criteria) being Model 2 (**Table 4, Step 2**).

*Dichotomous Outcomes:* For the two outcomes (bradykinesia and rigidity), too few persons had parkinsonian finding for ordinary regression to be appropriate. To obtain a valid analysis, we therefore employed a dichotomous measure, set to 0 if the finding was absent and to 1 when present. Our approach for these outcomes was similar to what was described above except that we employed logistic regressions rather than linear regressions. Since residuals from binary models are not suitable intermediate variables, we used another method to adjust the binary variables for age and sex. We fitted the logistic regression of the binary variable on age and sex, obtaining coefficients which we kept constant in all models for that outcome,. That is, all models for subtask scores and gait scores included terms for age and sex with the coefficients set equal to the age/sex coefficients. These terms are referred to as “offset” terms in the documentation of statistical software. When we employed standard backwards elimination with dichotomous outcomes the models (p-to-remove was 0.10), and included offset terms for age and sex, terms which were not eligible for elimination. As we described above, using the regression model obtained when the backwise elimination stopped, we calculated the estimated logit term for each mobility subtask. (Since the offset terms will be the same for the next step, we did not include the contribution of the offset term to the fitted logits.) As described above, step 1 in Stage 2, provides a check of our work, in that the regression coefficient in the linear logistic equation must be 1 if our calculations are correct. Since we present these models in terms of odds ratio, our tables show exp (1) =2.718 as the values of the coefficients for individual fitted mobility subtasks for dichotomous outcomes. This explains why the coefficients are 2.718 in Table 2 for bradykinesia and tremor which were based on logistic regressions and had only 1 subtask measure associated with the outcome of interest (**Table 2**)

*Cross Validation Study:* Using the same cases to construct summary measures and to measure their validity, can bias our estimates of accuracy. However, our approach does not favor one measure over another, so we can address our questions about which measures have large effects without suspecting that new results will overturn our findings. Nonetheless, to cross-validate our results, we applied the approach described to a 2^nd^ group of MAP participants [N=258; 78.3% women; Age (mean , 79.7 yrs SD=8.16 yrs) and Education (mean 15.0 yrs., SD=3.53 yrs.)]. We found that the major conclusions remain consistent. Some models even have larger adjusted R-squared values than in our initial group. However, the same three gait tasks (walk, sit to stand (S1) and turn) were identified as in our original data, coefficients are broadly similar, and all have the same signs (**Table S6**).

**APPENDIX S1 REFERENCES**

1. Bennett DA, Shannon KM, Beckett LA, Goetz CG, Wilson RS (1997) Metric properties of nurses' ratings of parkinsonian signs with a modified Unified Parkinson's Disease Rating Scale. Neurology 49: 1580-1587.

2. Bennett DA, Shannon KM, Beckett LA, Wilson RS (1999) Dimensionality of parkinsonian signs in aging and Alzheimer's disease. J Gerontol A Biol Sci Med Sci 54: M191-196.

3. Buchman AS, Leurgans SE, Boyle PA, Schneider JA, Arnold SE, et al. (2011) Combinations of Motor Measures More Strongly Predict Adverse Health Outcomes in Old Age: The Rush Memory and Aging Project, a Community-Based Cohort Study. BMC Medicine 9: 42.

4. Buracchio T, Arvanitakis Z, Leurgans S, Bennett DA (2010) Parkinsonian Signs And Incident Falls In Older Persons Without Parkinson's Disease. Journal of the American Geriatrics Society 58: 205-206.

5. Wilson RS, Bennett DA, Gilley DW, Beckett LA, Schneider JA, et al. (2000) Progression of Parkinsonism and Loss of Cognitive Function in Alzheimer Disease. Archives of Neurology 57: 855-860.

6. Wilson RS, Schneider JA, Beckett LA, Evans DA, Bennett DA (2002) Progression of gait disorder and rigidity and risk of death in older persons. Neurology 58: 1815-1819.

7. Wilson RS, Schneider JA, Bienias JL, Evans DA, Bennett DA (2003) Parkinsonianlike signs and risk of incident Alzheimer disease in older persons. Arch Neurol 60: 539-544.

8. Louis ED, Bennett DA (2007) Mild Parkinsonian signs: An overview of an emerging concept. Movement Disorders 22: 1681-1688.

9. Zhou G, Duan L, Sun F, Yan B, Ren S (2010) Association between mild parkinsonian signs and mortality in an elderly male cohort in China. Journal of Clinical Neuroscience 17: 173-176.

10. Buchman AS, Shulman JM, Nag S, Leurgans SE, Arnold SE, et al. (2012) Nigral pathology and parkinsonian signs in elders without Parkinson disease. Ann Neurol 71: 258-266.

11. Buchman AS, Leurgans SE, Nag S, Bennett DA, Schneider JA (2011) Cerebrovascular Disease Pathology and Parkinsonian Signs in Old Age. Stroke 42: 3183-3189.

12. Buchman AS, Nag S, Shulman JM, Lim AS, VanderHorst VG, et al. (2012) Locus coeruleus neuron density and parkinsonism in older adults without Parkinson's disease. Mov Disord 27: 1625-1631.

13. Evans AL, Duncan G, Gilchrist W (1991) Recording accelerations in body movements. Med Biol Eng Comput 29: 102-104.

14. Moe-Nilssen R (1998) A new method for evaluating motor control in gait under real-life environmental conditions. Part 2: Gait analysis. Clinical Biomechanics 13: 328-335.

15. Baltadjieva R, Giladi N, Gruendlinger L, Peretz C, Hausdorff JM (2006) Marked alterations in the gait timing and rhythmicity of patients with de novo Parkinson's disease. Eur J Neurosci 24: 1815-1820.

16. Hausdorff JM, Cudkowicz ME, Firtion R, Wei JY, Goldberger AL (1998) Gait variability and basal ganglia disorders: stride-to-stride variations of gait cycle timing in Parkinson's disease and Huntington's disease. Mov Disord 13: 428-437.

17. Hausdorff JM, Rios DA, Edelberg HK (2001) Gait variability and fall risk in community-living older adults: a 1-year prospective study. Arch Phys Med Rehabil 82: 1050-1056.

18. Schaafsma JD, Giladi N, Balash Y, Bartels AL, Gurevich T, et al. (2003) Gait dynamics in Parkinson's disease: relationship to Parkinsonian features, falls and response to levodopa. J Neurol Sci 212: 47-53.

19. Weiss A, Herman T, Plotnik M, Brozgol M, Giladi N, et al. (2011) An instrumented timed up and go: the added value of an accelerometer for identifying fall risk in idiopathic fallers. Physiol Meas 32: 2003-2018.

20. Weiss A, Mirelman A, Buchman AS, Bennett DA, Hausdorff JM (2013) Using a Body-Fixed Sensor to Identify Subclinical Gait Difficulties in Older Adults with IADL Disability: Maximizing the Output of the Timed Up and Go. PLoS ONE 8: e68885.

21. Weiss A, Herman T, Plotnik M, Brozgol M, Maidan I, et al. (2010) Can an accelerometer enhance the utility of the Timed Up & Go Test when evaluating patients with Parkinson's disease? Med Eng Phys 32: 119-125.

22. Mancini M, Horak FB, Zampieri C, Carlson-Kuhta P, Nutt JG, et al. (2011) Trunk accelerometry reveals postural instability in untreated Parkinson's disease. Parkinsonism Relat Disord 17: 557-562.

23. Prieto TE, Myklebust JB, Hoffmann RG, Lovett EG, Myklebust BM (1996) Measures of postural steadiness: differences between healthy young and elderly adults. IEEE Trans Biomed Eng 43: 956-966.

24. Rocchi L, Chiari L, Cappello A (2004) Feature selection of stabilometric parameters based on principal component analysis. Med Biol Eng Comput 42: 71-79.

25. Moe-Nilssen R, Helbostad JL (2004) Estimation of gait cycle characteristics by trunk accelerometry. Journal of Biomechanics 37: 121-126.
