# Supplementary material for: Associations between Quantitative Mobility Measures Derived from Components of Conventional Mobility Testing and Parkinsonian Gait in Older Adults
Source: PLoS One. 2014 Jan 22;9(1):e86262. doi: 10.1371/journal.pone.0086262 (PMC3899223; doi:10.1371/journal.pone.0086262)
Supplement: Table S1 — Modified UPDRS Assessment. (DOCX) [file pone.0086262.s002.docx]

**Table S1. Modified UPDRS Assessment**

| **Doman/Tasks** |
| --- |
| **Parkinsonian Gait** |
| Turning |
| Posture |
| Postural stability |
| Arising from a chair |
| Shuffling gait |
| Body bradykinesia |
| **Rigidity** |
| Neck |
| Right arm |
| Left arm |
| Right leg |
| Left leg |
| **Bradykinesia** |
| Right finger tap |
| Left finger tap |
| Right fist clench |
| Left fist clench |
| Right pronation-supination |
| Left pronation-supination |
| Right leg agility |
| Left leg agility |
| **Tremor** |
| Right arm rest tremor |
| Left arm rest tremor |
| Right leg rest tremor |
| Left leg rest tremor |
| Chin-jaw rest tremor |
| Right arm action-postural tremor |
| Left arm action-postural tremor |

In prior publications, four previously established parkinsonian sign scores were derived from the 26 items assessed. Each of the 26 items was rated on a 0-5 scale: **0**=*normal*; **1**=*slowing or reduction in amplitude which could be normal*; **2**= *mild slowing and reduction in amplitude*; **3**=*moderately impaired* *with definite early fatiguing and may have occasional arrests in movement*; **4**=*severely impaired with frequent hesitation in initiating movements*; **5**= *can barely perform the task*.
