# Supplementary material for: Associations between Quantitative Mobility Measures Derived from Components of Conventional Mobility Testing and Parkinsonian Gait in Older Adults
Source: PLoS One. 2014 Jan 22;9(1):e86262. doi: 10.1371/journal.pone.0086262 (PMC3899223; doi:10.1371/journal.pone.0086262)
Supplement: Table S2 — Gait Measures and Gait Scores Derived from Whole Body Sensor Recordings. (DOCX) [file pone.0086262.s003.docx]

**Table S2. Gait Measures and Gait Scores Derived from Whole Body Sensor Recordings**

| **MOBILITY SUBTASKS** | **GAIT MEASURES*** | | | **GAIT SCORES *** | | |
| --- | --- | --- | --- | --- | --- | --- |
|  | **Measure (units)** | **Mean (SD)** | **Q1, Q3** | **Score** | **Mean** | **Range** |
| **WALK** | Speed (m/s) | 0.41 (0.09) | 0.35, 0.48 | **Speed** | 0.07(1.00) | -2.35, 2.30 |
|  | Stride length (m) | 0.79 (0.19) | 0.65,0.89 |  |  |  |
|  | Cadence (steps/min) | 64.02 (7.22) | 29.40, 33.89 | **Cadence** | 1.18 (1.00) | 0.0001, 5.52 |
|  | Stride time CV (%) | 4.18 (2.35) | 2.73, 4.77 | **Variability** | 0.07 (1.00) | -1.03, 6.35 |
|  | Stride regularity [g^2^] | 0.31 (0.11) | 0.23, 0.38 | **Regularity** | 0.06 (1.00) | -2.73, 2.79 |
|  | Step symmetry | 1.31 (0.36) | 1.11, 1.50 |  |  |  |
| **SIT TO STAND (S1)** | AP Duration (s) | 0.92 (0.19) | 0.78, 1.03 | **Ant-Post** | -0.04(1.00) | -3.14, 2.62 |
|  | AP Jerk (g/s) | -1.21 (0.69) | -3-1.61, -1.08 |  |  |  |
|  | AP range(g) | 0.95 (0.11) | 0.88, 1.02 | **Range** | 0.01 (1.00) | -1.96, 6.37 |
|  | AP Acc SD (g) | 0.26 (0.07) | 0.22, 0.31 |  |  |  |
|  | Pitch range (deg/s) | 9.00 (1.07) | 8.20, 9.06 |  |  |  |
|  | Pitch jerk (deg/s^2^) | 199.74 (97.60) | 130.49, 252.86 | **Posterior** | 0.11 (1.00) | -2.85, 3.02 |
|  | Median (deg/s) | -0.13 (0.19) | -0.26, 0.02 |  |  |  |
|  | Pitch Duration (s) | 0.92 (0.13) | 0.90, 1.10 |  |  |  |
| **STAND TO SIT (S2)** | Pitch jerk (deg/s^2^) | 151.33 (81.67) | 92.92, 192.48 | **Jerk** | - 0.01(1.00) | - 2.30, 4.01 |
|  | AP duration (s) | 0.96 (0.16) | 0.86, 1.06 |  |  |  |
|  | Pitch duration (s) | 1.00 (0.15) | 0.83, 0.99 |  |  |  |
|  | AP Jerk (g/s) | 1.06 (0.55) | 0.73, 1.24 |  |  |  |
|  | AP range (g) | 0.99 (0.10) | 0.92, 1.06 | **Range** | -0.12 (1.00) | -2.75, 2.32 |
|  | Pitch range (deg/s) | 8.81 (0.97) | 8.20, 9.71 |  |  |  |
|  | AP Acc. SD (g) | 0.28 (0.06) | 0.23, 0.32 |  |  |  |
|  | Median (g) | -0.16 (0.19) | -0.32, -0.01 | **Median** | 0.01 (1.00) | -3.06, 2.74 |
| **TURNING** | Yaw, Turn 1 (deg/s) | 149.48 (38.45) | 122.89, 147.00 | **Yaw** | 0.09 (1.00) | -3.99, 2.15 |
|  | Yaw, Turn 2 (deg/s) | 148.86 (40.24) | 118.15, 176.10 |  |  |  |
|  | Duration, Turn 1 (s) | 2.21 (0.52) | 1.86, 2.47, |  |  |  |
|  | Duration, Turn 2 (s) | 2.12 (0.52) | 1.75, 2.41 |  |  |  |
|  | Frequency, Turn 1 (Hz) | 1.93 (0.43) | 1.56, 2.25 |  |  |  |
|  | Frequency, Turn 2 (Hz) | 1.72 (0.54) | 1.37, 2.05 | **Frequency** | -0.04 (1.00) | -2.38, 2.55 |
| **STANDING POSTURE** | Jerk [g/s]^2^ | -3.63 (0.53 | -3.99, -3.31 | **Sway** | 0.001 (1.00) | -4.36, 2.13 |
|  | RMS distance [g] | -1.86 (0.23) | -2.04, -1.74 |  |  |  |
|  | Total power [psd] | -1.28 (0.63) | -4.43, -3.71 |  |  |  |
