# Supplementary material for: Associations between Quantitative Mobility Measures Derived from Components of Conventional Mobility Testing and Parkinsonian Gait in Older Adults
Source: PLoS One. 2014 Jan 22;9(1):e86262. doi: 10.1371/journal.pone.0086262 (PMC3899223; doi:10.1371/journal.pone.0086262)
Supplement: Table S3 — Association of Gait Scores with Age and Sex. (DOCX) [file pone.0086262.s004.docx]

**Table S3. Association of Gait Scores with Age and Sex**

| **Mobility Subtasks** | **Gait Scores** | **R_adj_^2^** | **Age** | **Sex** |
| --- | --- | --- | --- | --- |
| **Walk** | **Speed** | 0.1492 | -0.052 (0.007,<0.001) | 0.258(0.111,0.021) |
|  | **Cadence** | -0.0036 | 0.007 (0.008, 0.387) | 0.012(0.131, 0.927) |
|  | **Variability** | 0.0189 | 0.020 (0.008,0.012) | -0.218(0.130, 0.094) |
|  | **Regularity** | 0.1070 | -0.021 (0.004,<0.001) | -0.177(0.062, 0.005) |
| **Sit to Stand (S1)** | **Anterior-Posterior** | 0.0443 | 0.030 (0.008, <0.001) | -0.004(0.131, 0.977) |
|  | **Range** | 0.0772 | -0.015 (0.003,<0.001) | 0.103(0.053,0.053) |
|  | **Posterior** | 0.1104 | -0.046 (0.008,<0.001) | 0.080(0.129, 0.536) |
| **Stand to Sit (S2)** | **Jerk** | 0.0165 | -0.006 (0.007, 0.399) | -0.282(0.118, 0.017) |
|  | **Range** | -0.0022 | 0.003 (0.007, 0.721) | 0.127(0.119, 0.287) |
|  | **Median** | -0.0054 | 0.003 (0.008, 0.770) | -0.086(0.141, 0.543) |
| **Turning** | **Yaw** | 0.1891 | -0.055 (0.007,<0.001) | 0.099(0.113, 0.383) |
|  | **Frequency** | -0.0066 | 0.003 (0.006, 0.685) | 0.008(0.106, 0.942) |
| **Standing Posture** | **Sway** | 0.0417 | -0.021 (0.007, 0.005) | -0.331(0.122, 0.007) |
