# Supplementary material for: Associations between Quantitative Mobility Measures Derived from Components of Conventional Mobility Testing and Parkinsonian Gait in Older Adults
Source: PLoS One. 2014 Jan 22;9(1):e86262. doi: 10.1371/journal.pone.0086262 (PMC3899223; doi:10.1371/journal.pone.0086262)
Supplement: Table S4 — Correlations of Walking, Turning and Sway Gait Scores. (DOCX) [file pone.0086262.s005.docx]

**Table S4. Correlations of Walking, Turning and Sway Gait Scores**

| **Gait Score** | **Speed** | **Cadence** | **Variability** | **Regularity** | **Yaw** | **Frequency** | **Sway** |
| --- | --- | --- | --- | --- | --- | --- | --- |
| **Speed** | **1.00** | 0.06 | -0.27^§^ | 0.45^§^ | 0.76^§^ | 0.09 | 0.13 |
| **Cadence** | 0.08 | **1.00** | 0.03 | 0.16^ | 0.04 | -0.03 | 0.02 |
| **Variability** | -0.24^§^ | 0.02 | **1.00** | -0.40^§^ | 0.19^*^ | -0.09 | -0.03 |
| **Regularity** | 0.41^§^ | 0.19* | -0.40^§^ | **1.00** | 0.44^§^ | 0.19* | 0.18^*^ |
| **Yaw** | 0.72^§^ | 0.07 | -0.14 | 0.43^§^ | **1.00** | 0.04 | 0.15 |
| **Frequency** | 0.12 | -0.04 | -0.11 | 0.22* | 0.12 | **1.00** | -0.03 |
| **Sway** | 0.12 | 0.03 | -0.03 | 0.12 | 0.13 | -0.03 | **1.00** |

Values above the diagonal are Pearson correlations; values below the diagonal are partial correlations (which can also be thought of as the correlation of the adjusted values.) ^§^p<0.001 ; *p<0.01; ^p<0.05
