# Supplementary material for: Associations between Quantitative Mobility Measures Derived from Components of Conventional Mobility Testing and Parkinsonian Gait in Older Adults
Source: PLoS One. 2014 Jan 22;9(1):e86262. doi: 10.1371/journal.pone.0086262 (PMC3899223; doi:10.1371/journal.pone.0086262)
Supplement: Table S5 — Correlations of Transition Scores, Turning and Sway Gait Scores. (DOCX) [file pone.0086262.s006.docx]

**Table S5. Correlations of Transition Scores, Turning and Sway Gait Scores**

| **Gait Score** | **Ant-Post (S1)** | **Range (S1)** | **Posterior (S1)** | **Jerk**  **(S2)** | **Range (S2)** | **Median (S2)** | **Yaw** | **Sway** |
| --- | --- | --- | --- | --- | --- | --- | --- | --- |
| **Ant-Post (S1)** | **1.00** | -0.07 | -0.47^§^ | -0.37^§^ | -0.18^*^ | -0.05 | -0.41^§^ | -0.01 |
| **Range (S1)** | -0.04 | **1.00** | 0.39^§^ | 0.16^^^ | 0.27^§^ | -0.02 | 0.26^§^ | 0.02 |
| **Posterior (S1)** | -0.44^§^ | 0.32^§^ | **1.00** | 0.44^§^ | 0.10 | -0.18^ | 0.50^§^ | 0.17^ |
| **Jerk (S2)** | -0.38^§^ | 0.22* | 0.47^§^ | **1.00** | 0.40^§^ | -0.16* | 0.36^§^ | 0.12 |
| **Range (S2)** | -0.18^ | 0.30^§^ | 0.10 | 0.47^§^ | **1.00** | -0.02 | 0.09 | 0.05 |
| **Median (S2)** | -0.01 | 0.007 | -0.19^ | -0.12 | 0.01 | **1.00** | 0.004 | -0.08 |
| **Yaw** | -0.33^§^ | 0.12 | 0.44^§^ | 0.39^§^ | 0.08 | 0.03 | **1.00** | 0.15 |
| **Sway** | 0.002 | -0.02 | 0.13 | 0.15^ | -0.04 | -0.10 | 0.13 | **1.00** |

Values above the diagonal are Pearson correlations; values below the diagonal are partial correlations (which can also be thought of as the correlation of the adjusted values.

^§^p<0.001 ; *p<0.01; ^p<0.05
