# Supplementary material for: Associations between Quantitative Mobility Measures Derived from Components of Conventional Mobility Testing and Parkinsonian Gait in Older Adults
Source: PLoS One. 2014 Jan 22;9(1):e86262. doi: 10.1371/journal.pone.0086262 (PMC3899223; doi:10.1371/journal.pone.0086262)
Supplement: Table S6 — Cross Validation of the Original Results in a 2nd Group of Older Adults (N = 258). (DOCX) [file pone.0086262.s007.docx]

**Table S6. Cross Validation of the Original Results in a 2^nd^ Group of Older Adults (N=258)**

|  | **STEP 1**  **Linear regression models** | | | | **STEP 2**  **Backward elimination** | |
| --- | --- | --- | --- | --- | --- | --- |
| **Mobility**  **Subtasks** | **Model A** | **Model B** | **Model C** | **Model D** | **Model 1** | **Model 2** |
| **Adj R-Sq** | 0.174 | 0.233 | 0.115 | 0.206 | 0.325 | 0.318 |
| **Walk** | 1.000 (0.137, <0.001) |  |  |  | 0.397 (0.149,0.008) | 0.420 (0.149,0.005) |
| **Sit-Stand (S1)** |  | 1.000 (0.113, <0.001) |  |  | 0.571 (0.127, <0.001) | 0.621 (0.125,<0.001) |
| **Stand-Sit (S2)** |  |  | 1.000 (0.170, <0.001) |  | 0.313 (0.166,0.061) |  |
| **Turning** |  |  |  | 1.000 (0.122, <0.001) | 0.441 (0.143,0.002) | 0.494 (0.141,<0.001) |

Cell entries include estimated regression parameter **β**, together with its estimated standard error and the corresponding p-value (SE, p-Value). For models A-D, the constructed variable is fitted value for the adjusted gait score based on the adjusted covariates selected. As an automatic consequence, when this constructed variable is itself used as the only independent variable, its regression coefficient is precisely 1. Model 1 (Step 2) is the starting model for the backwards elimination phase, in which every constructed variable with p<0.05 (here, 4 mobility subtasks) are included. In the 2^nd^ group of participants used for this cross-validation study, the sway subtask was not included, since the model with the sway subtask by itself showed only a trend for significance with parkinsonian gait (Estimate 1.000, S.E 0.542, p=0.066). The backwards elimination phase (Step 2) then ran twice. Using a SAS default settings, p-to-remove=0.10, it stopped at Model 2, in which 3 of the 5 mobility subtasks remain significant. These are the same subtasks which were identified in our original data as shown in the text (Table 4).
